# Long-term athletic training does not alter age-associated reductions of left-ventricular mid-diastolic lengthening or expansion at rest

**DOI:** 10.1007/s00421-020-04418-1

**Published:** 2020-07-04

**Authors:** Alexander Beaumont, Amy Campbell, Viswanath Unnithan, Fergal Grace, Allan Knox, Nicholas Sculthorpe

**Affiliations:** 1 School of Sport, York St. John University, York, UK; 2 Institute of Clinical Exercise and Health Sciences, School of Science and Sport, University of the West of Scotland, Stephenson Place, Hamilton International Technology Park Blantyre, Glasgow, UK; 3 Faculty of Health, School of Health Science and Psychology, Federation University Australia, Ballarat, VIC Australia; 4 Exercise Science Department, California Lutheran University, Thousand Oaks, CA USA

**Keywords:** Ageing, Mechanics, Exercise, Speckle-tracking echocardiography, Ventricular strain

## Abstract

**Purpose:**

The interaction of ageing and exercise training status on left-ventricular (LV) peak strain is unclear. Additionally, strain analysis across the entire cardiac cycle facilitates a more detailed assessment of deformation, yet this has not been implemented to characterize the ageing LV and in association with training status. This study investigated healthy ageing and training status on LV systolic and diastolic strain utilizing novel echocardiographic applications.

**Methods:**

Forty healthy males were included and allocated into four groups; young recreationally active (Y_RA,_ *n* = 9; 28 ± 5 years), old recreationally active (O_RA_, *n* = 10; 68 ± 6), young trained (Y_T,_ *n* = 10; 27 ± 6 years), and old trained (O_T_, *n* = 11, 64 ± 4 years) groups. Two-dimensional speckle-tracking echocardiography was performed to ascertain peak LV longitudinal and circumferential strain (base and apex) strain within each myocardial layer and at 5% increments across the cardiac cycle.

**Results:**

Older groups had lower diastolic longitudinal lengthening and circumferential expansion between 40–85% mid-diastole, regardless of training status (*P* < 0.05). Whereas, strain throughout systole was similar between groups (*P* > 0.05). Longitudinal and circumferential (base and apex) peak and layer-specific strain did not differ between groups (*P* > 0.05).

**Conclusion:**

Novel applications of diastolic strain revealed lower age-associated LV longitudinal lengthening and circumferential expansion in older age. Yet, diastolic strain profiles did not differ based on chronic habits of exercise training and, thus, older trained men did not demonstrate an attenuation of age-associated differences in mid-diastolic LV strain.

**Electronic supplementary material:**

The online version of this article (10.1007/s00421-020-04418-1) contains supplementary material, which is available to authorized users.

## Introduction

The left ventricle (LV) is characterized by structural and functional modifications with advancing age, including increased wall thickness and reductions in diastolic function (Lakatta and Levy 2003). Resting systolic function, however, as determined by ejection fraction, remains well preserved with healthy ageing (Lakatta and Levy 2003). However, ejection fraction only provides an indirect measure of contractile function and is a surrogate marker describing LV pump function (Blessberger and Binder 2010). The development of speckle tracking echocardiography (STE) has facilitated a direct assessment of myocardial tissue deformation (Mor-Avi et al. 2011). Introduced some 15 years ago (Leitman et al. 2004), its application within clinical and research settings is expanding to assess LV shortening and lengthening by quantifying strain and strain rate (SR) (Mor-Avi et al. 2011; Lang et al. 2015).

Studies have identified that with healthy ageing, longitudinal strain may decline (Dalen et al. 2010; Sun et al. 2013; Hung et al. 2017; Alcidi et al. 2018) or remain relatively constant (Yingchoncharoen et al. 2013; Kocabay et al. 2014; Nagata et al. 2017). Similarly, circumferential strain may be maintained (Zghal et al. 2011; Nagata et al. 2017) or even increase with ageing (Sun et al. 2013; Hung et al. 2017). Strain is not homogenous across the LV wall, however, but is highest in the endocardium and lowest in the epicardium (Abou et al. 2017). The most recent versions of STE software enable the identification of strain within three myocardial layers to ascertain mechanical (dys) function (Sharif et al. 2018), through layer-specific analysis. Although the influence of ageing on longitudinal strain is debated, the transmural gradient (endocardium-to-epicardium difference/ratio) appears to be preserved with advancing age (Nagata et al. 2017; Alcidi et al. 2018).

Whether LV strain profiles of chronic endurance exercisers differ to their non-athletic counterparts is unclear and thus, it is unknown whether chronic exercise training can attenuate age-associated differences in LV strain. Through meta-analysis, longitudinal strain was similar between age-matched older athletic and non-athletic males (Beaumont et al. 2018), albeit only five studies were available for inclusion, which were significantly heterogeneous. Studies that have also included younger untrained cohorts, to enable age and training-related assessments within the same investigation, are also conflicting. Howden et al. (2017) reported that master athletes attenuated the lower longitudinal strain, whereas others observed no age- or training-associated effect on longitudinal or circumferential strain (Donal et al. 2011). Moreover, lower longitudinal strain in athletes than controls has also been noted, regardless of age (Maufrais et al. 2017).

Furthermore, while identification of peak values is important, strain analysis across the entire cardiac cycle facilitates a more nuanced assessment of deformation including key phases that are not represented in peak data. This technique has been introduced to clinical and athletic populations (Utomi et al. 2014; Sharif et al. 2018; Johnson et al. 2018), but has not been used to characterize the ageing LV in association with training status. When assessed using expensive and invasive end-diastolic volume–pressure curves, LV compliance, and distensibility progressively decrease with senescence (Fujimoto et al. 2012), but this may be preserved in master athletes (Arbab-Zadeh et al. 2004). However, similar data concerning rapidly and non-invasively derived STE strain across the cardiac cycle in athletic and ageing cohorts have not been documented.

Together, more data are required pertaining to the influence of ageing and exercise training on peak strain. Additionally, novel strain techniques to differentiate myocardial layers and at progressive increments across the entire cardiac cycle are warranted in such populations. Accordingly, this study aimed to investigate the influence of healthy ageing and exercise training on systolic and diastolic LV strain, yet in the absence of cardiovascular diseases and treatment medications to isolate ‘true’ ageing and exercise training from confounding factors. It was hypothesized that age-associated reductions in longitudinal systolic and diastolic strain would be mitigated by chronic athletic training to similar levels as younger, recreationally active (RA) males.

## Materials and methods

### Participant selection and identification of healthy controls

This cross-sectional study required participants to visit the laboratory on two separate occasions having been asked to abstain from alcohol, caffeine (24 h), and vigorous exercise (48 h) prior to both visits. The study was granted ethical approval and all participants provided written, informed consent before being enrolled.

We aimed to recruit two adults with physiologically meaningful differences in age; therefore, we recruited young adults between the ages of 18–40 years (final age range 18–36yrs) and old individuals 55–80 years (final age range 59–80 years; see Table 1). In total, sixty–eight males were recruited and divided into one of four groups: young recreationally active (Y_RA_), young trained (Y_T_), old recreationally active (O_RA_), and old trained (O_T_). Individuals were approached at local sports clubs (running, cycling, and triathlon), via social media posts, through word-of-mouth, attendance at social clubs (i.e., chess and bowls clubs) and also at local community events, such as ‘Men’s Shed’ and ‘University of the Third Age (U3A)’.

**Table 1:** Baseline physical and exercise characteristics including training habits and maximal oxygen uptake in young recreationally active (Y_RA_), young trained (Y_T_), old recreationally active (O_RA_), and old trained (O_T_) participants

	Young		Old		*P* value		
	Recreationally active (Y_RA_)	Trained (Y_T_)	Recreationally active (O_RA_)	Trained (O_T_)	Age	Training	Interaction
Number of participants	9	10	10	11			
Age (years)	28 ± 5 (21–35)	27 ± 6 (18–36)	68 ± 6 (60–80)	64 ± 4 (59–70)	** < 0.001**	0.21	0.46
Exercise characteristics							
Minutes per week	67 ± 87	450 ± 239	63 ± 67	540 ± 180	0.41	** < 0.001**	0.37
Training years	–	5 ± 4	–	34 ± 14			
Training start age (years)	–	23 ± 8	–	31 ± 11			
Height (cm)	177 ± 4	180 ± 8	175 ± 8	173 ± 4	**0.02**	0.65	0.31
Mass (kg)	80.5 ± 7.8	75.0 ± 9.3	82.0 ± 20.2	67.0 ± 5.6	0.40	**0.01**	0.22
Systolic blood pressure (mmHg)	131 ± 9	126 ± 10	131 ± 11	132 ± 16	0.47	0.63	0.34
Diastolic blood pressure (mmHg)	77 ± 8	68 ± 7	74 ± 8	74 ± 8	0.47	0.09	0.06
Mean arterial pressure (mmHg)	95 ± 8	87 ± 7	93 ± 8	94 ± 10	0.38	0.18	0.11
Resting heart rate (beats^.^min^−1^)	61 ± 11	49 ± 11	54 ± 5	47 ± 7	0.14	**0.001**	0.35
Rate pressure product (mmHg/beats^.^min^−1^)	8049 ± 1810	6084 ± 1063	7038 ± 571	6231 ± 1073	0.26	** < 0.001**	0.13
Body surface area (m^2^)	1.99 ± 0.11	1.94 ± 0.15	1.98 ± 0.27	1.79 ± 0.09	0.17	**0.03**	0.22
Body mass index (kg^.^m^2^)	25.5 ± 1.7	23.1 ± 2.2	26.8 ± 5.1	22.3 ± 1.6	0.83	** < 0.001**	0.31
$\dot{\mathrm{V}}{\mathrm{O}}_{2\mathrm{m}\mathrm{a}\mathrm{x}}$ (L^.^min^−1^)	3.9 ± 0.4	4.8 ± 0.7	2.7 ± 0.4	3.4 ± 0.3	** < 0.001**	** < 0.001**	0.40
$\dot{\mathrm{V}}{\mathrm{O}}_{2\mathrm{m}\mathrm{a}\mathrm{x}}$ (mL^.^kg^−1.^min^−1^)	48.5 ± 5.0	64.1 ± 7.7	34.9 ± 7.3	50.1 ± 3.6	** < 0.001**	** < 0.001**	0.93
Age predicted $\dot{\mathrm{V}}{\mathrm{O}}_{2\mathrm{m}\mathrm{a}\mathrm{x}}$ (%)	92 ± 9	121 ± 12	94 ± 21	128 ± 8	0.32	** < 0.001**	0.55

$\dot{\mathrm{V}}{\mathrm{O}}_{2\mathrm{m}\mathrm{a}\mathrm{x}}$, maximal oxygen uptake

Data are means +/- SD. Significant main effects are highlighted in bold

*P* ≤ 0.050. vs Y_RA_; † vs. Y_T_; ‡ vs. O_RA_

Medical questionnaires assessed suitability for healthy individuals, with 12 older individuals (O_RA_, *n* = 9 and O_T_, *n* = 3) excluded based on treatment for cardiovascular diseases (angina, myocardial infarction, peripheral vascular disease, and stroke), type 2 diabetes mellitus, use of cardioactive medications for treatment [i.e., anti-hypertensives (calcium channel blockers) and beta-blockers] or blood pressure classified as ‘hypertensive crisis’ (≥ 180 systolic or 120 diastolic mmHg). Five individuals withdrew due to personal reasons (Y_T_, *n* = 2; O_RA_, *n* = 1; and O_T_, *n* = 2), with two Y_RA_ participants excluded due to violations of requirements prior to or during data collection, with a further two Y_RA_ participants excluded due to being smokers. All remaining participants were not habitual smokers at the time of assessment.

A self-reported questionnaire was used to ascertain the number of hours spent performing aerobic exercise, corresponding to high dynamic activities (Mitchell et al. 2005). This model was used to quantify exercise dosage but not used to define trained and RA groups according to specific components. While two Y_RA_ participants competed in recreational team-based sports, (soccer and field hockey), all reported non-structured exercise habits and took part in < 2 h per week of continuous endurance exercise (running, cycling, and rowing). Similarly, some O_RA_ individuals performed recreational hill walking, golf, walking soccer, and gym-based exercise, albeit all ≤ 2 h per week of continuous endurance exercise.

Y_T_ were required to train > 6 months to avoid an acutely elevated increase in LV mechanics (Aksakal et al. 2013), and were required to be involved with competition (Beaumont et al. 2016). A continuation of training and absence of injury between laboratory visits was required; therefore, three Y_T_ participants were excluded. O_T_ commenced exercise before 64 years of age, based on prior work highlighting the age-associated profile of LV stiffness, and a threshold of adaptation in compliance (Fujimoto et al. 2010, 2012; Howden et al. 2018). Also O_T_ were required to be training ≥ 5 year (Donal et al. 2011; Matelot et al. 2016), together leading to four O_T_ being excluded. Trained individuals were not professional athletes, but were all involved with competitive athletic events from regional to international level. Y_T_ participants engaged with running only (*n* = 3), cycling only (*n* = 3), or both modalities (*n* = 4). Similarly, O_T_ individuals took part in running only (*n* = 5), cycling only, (*n* = 2) or both modalities (*n* = 4). Three OT previously competed internationally, including one a past half marathon world champion for their respective age group. See supplementary material 1 for Y_T_ and O_T_ training durations, volumes, and dose. Twenty-eight individuals were excluded, resulting in 40 males included in the final analysis (Y_RA_, *n* = 9; Y_T_, *n* = 10; O_RA_, *n* = 10, and O_T_, *n* = 11).

### Protocol and experimental procedures

During visit 1, unshod stature and body mass were measured using a free-standing stadiometer, (Harpenden, HAR-98.602) and scales (877, Seca, Germany). Body mass index (BMI) was calculated as*:* $\frac{\mathrm{B}\mathrm{o}\mathrm{d}\mathrm{y} \mathrm{m}\mathrm{a}\mathrm{s}\mathrm{s} (\mathrm{k}\mathrm{g})}{\mathrm{S}\mathrm{t}\mathrm{a}\mathrm{t}\mathrm{u}\mathrm{r}\mathrm{e} {(\mathrm{m})}^{2}}$ and body surface area (BSA) determined using the Mosteller formula (Mosteller 1987), $\surd \frac{ \mathrm{S}\mathrm{t}\mathrm{a}\mathrm{t}\mathrm{u}\mathrm{r}\mathrm{e} \left(\mathrm{c}\mathrm{m}\right) \times \mathrm{B}\mathrm{o}\mathrm{d}\mathrm{y} \mathrm{m}\mathrm{a}\mathrm{s}\mathrm{s} (\mathrm{k}\mathrm{g})}{3600}$. The Ekblom Bak submaximal test was used to predict maximal oxygen uptake ($\dot{\mathrm{V}}{\mathrm{O}}_{2\mathrm{m}\mathrm{a}\mathrm{x}}$) (Björkman et al. 2016), using an electronically braked cycle ergometer (Lode, Excalibur, Groningen, The Netherlands). Both absolute and relative $\dot{\mathrm{V}}{\mathrm{O}}_{2\mathrm{m}\mathrm{a}\mathrm{x}}$ were recorded and relative was compared with normative values reported by the HUNT fitness study (Loe et al. 2013) at the individual level to determine the % age-predicted $\dot{\mathrm{V}}{\mathrm{O}}_{2\mathrm{m}\mathrm{a}\mathrm{x}}$.

During visit 2, following 5 min’ supine rest, three blood pressure readings, using an automated sphygmomanometer (OMRON, 705IT, Hoofddorp, The Netherlands), were recorded each separated by 2 min. A resting echocardiographic assessment was conducted with resting HR recorded offline from the three-lead electrocardiogram (ECG) inherent to the ultrasound machine, as the average of cardiac cycles used for LV outflow tract. Mean arterial pressure (MAP) was calculated as: $\frac{(\mathrm{S}\mathrm{B}\mathrm{P}+2 \times \mathrm{D}\mathrm{B}\mathrm{P})}{3}$ and rate pressure product (RPP) as: HR x SBP (Weippert et al. 2013).

### Echocardiography

Resting echocardiographic assessment was undertaken in the left-lateral decubitus position using a commercially available ultrasound (Vivid iq, GE Medical, London) with a phased array transducer (3S, 1.4–3.8 MHz; M5Sc-RS, 1.5–4.6 MHz). Images were acquired and analyzed by a single sonographer (AB) in accordance with standard procedures (Maragiannis and Nagueh 2015; Lang et al. 2015). Using the parasternal long-axis view, the assessment of end-diastolic LV structure and dimensions included interventricular septal (IVS) and posterior (PWT) wall thickness and end-diastolic diameter (LVEDD) using the two-dimensional B-mode image. LV length was determined as the greatest base-to-apex length from either apical 4 or 2-chamber view (Lang et al. 2015). LVM was calculated according to current recommendations (Lang et al. 2015). The quantification of LV geometry included relative wall thickness (RWT), concentricity indexed to LV end-diastolic volume (LVEDV) (LVEDV^0.667^), and sphericity index as the ratio between LVEDD and LV length (van Dalen et al. 2009). LV stroke volume (SV) was calculated as the product of LV outflow tract cross-sectional area, determined from the parasternal long axis, and velocity–time integral (VTI) trace obtained from apical five-chamber outflow tract. LVM was scaled directly to BSA (Lang et al. 2015), LV dimensions were indexed to BSA^0.5^ and volumes to BSA^1.5^ (Oxborough et al. 2016; Forsythe et al. 2018b).

E-wave and A-wave velocities were determined in the apical four-chamber view at the tip of the mitral valve leaflets and their ratio (E/A) calculated. Longitudinal tissue velocities were established using pulsed waved Doppler in the apical four-chamber view from medial and lateral sites, and then averaged. Unidimensional velocities from the septal and lateral regions during systole (*s*′), early (*e*′) and late (a’) diastole were averaged, and then, their ratio (*e*′/*a*′) calculated. Doppler tissue velocities were then scaled to LV length (Batterham et al. 2008) and LV filling pressure was estimated as *E*/*e*′.

### Speckle tracking echocardiography

Peak LV longitudinal mechanics were determined using the apical 4- and 2-chamber views. The parasternal short-axis images at the basal and apical levels were acquired as proximally and caudally, respectively, as possible. The basal plane was obtained when circular at the level of the full mitral valve in the absence of distally visible chordae tendineae whilst avoiding the aortic valve during basal systolic excursion. The apex was captured as distally as possible without the visibility of papillary muscles (van Dalen et al. 2008) by tilting the transducer from an original apical 4-chamber orientation and moved slightly to the point above LV luminal obliteration (Mor-Avi et al. 2011). Thus, regarding the apex image, the most caudal cardiac cycle with the smallest LV chamber at end-systole was selected for speckle-tracking analysis. See supplementary material 2 for example images.

Images were recorded at frame rates ~ 71 fps and were analyzed offline using dedicated semi-automated software (EchoPac, version 202). Aortic valve closure (AVC) was identified as end-systolic timing from the pulsed wave tracings obtained from apical 5-chamber LV outflow tract. The clearest cardiac cycle with most optimized myocardial layer delineation was selected, and then, the endocardial border was manually traced before an automated region of interest (ROI) was generated to encompass the entirety of the LV wall extending to the epicardial border. The operator manually adjusted the ROI paying attention to ensuring LV and RV trabeculations, moderator band, papillary muscles, and pericardium were not included within the ROI. The software automatically divided the myocardial wall into six segments and in the instance that two or more segments could not be tracked sufficiently, the image was excluded from analysis. Raw files were exported from the EchoPac software and imported into custom software (Automated Strain Analysis v1, Glasgow, UK). The custom software applied a 500-point cubic spline to each of the systolic and diastolic portions of the cardiac cycle (derived from AVC). The splined data were then used to identify systolic and diastolic peaks and relative timings, as well as identifying strain or strain rate values at 5% increments of the systolic and diastolic phases, respectively.

Systolic SR (SR_S_) was identified as the peak of the SR-time curve and for early diastolic SR (SR_E_), a filter of 50 ms from AVC was used to exclude peaks in isovolumic relaxation time. Using the ECG, P-wave timing was identified and the late diastolic SR (SR_A_) was determined after this detection point. Therefore, peak SR_S_, SR_E_, and SR_A_ were determined in the longitudinal orientation and from the parasternal short-axis views for radial and circumferential strains at the basal and apical levels and their averages calculated.

Within-day, test–retest reproducibility of STE-derived strain and SR for the sonographer of this study (AB) was conducted in a subset of eight, young males. For peak indices relating to this study, the coefficients of variations are as follows: four-chamber longitudinal strain, 2.9%; basal circumferential strain, 10.8%; apical circumferential strain, 4.6%. See supplementary material 3 for extended reproducibility including normalized analysis across the cardiac cycle.

### Statistical analysis

For all variables, a two-way analysis of variance (ANOVA) was used to determine an age effect, a training effect, and their interaction (age × training). In the presence of a statistically significant interaction effect, a Tukey’s adjusted post hoc test was conducted for multiple pairwise comparisons. Similarly, for analysis of the entire cardiac cycle, 5% increments were individually assessed using two-way ANOVA, with significant interactions explored further using Tukey’s post hoc test. Post hoc power calculation based on LVM indexed to BSA (a known marker for physiological adaption to exercise) identified an achieved statistical power of 99%. Statistical significance was granted at *P* ≤ 0.050 and analyses performed using jamovi [version 1.1.9 (The jamovi project 2019)].

## Results

### Baseline anthropometrics and exercise characteristics

Baseline characteristics of the four groups are presented in Table 1. Trained and RA cohorts were well matched for age. Older groups were shorter than younger cohorts, but height did not differ between trained and RA. Body mass, BMI, and BSA did not differ between ages; however, all were lower in trained than RA. No age or training effects were noted for systolic, diastolic, or mean arterial blood pressures. No age differences were observed for HR or RPP, whereas trained had lower HR and RPP than RA. Trained cohorts performed more minutes of exercise per week than RA, with an unremarkable difference between ages. Absolute and relative $\dot{\mathrm{V}}{\mathrm{O}}_{2\mathrm{m}\mathrm{a}\mathrm{x}}$ was lower in older than younger cohorts; however, trained groups had higher $\dot{\mathrm{V}}{\mathrm{O}}_{2\mathrm{m}\mathrm{a}\mathrm{x}}$ than their RA counterparts. Trained cohorts had higher age-predicted $\dot{\mathrm{V}}{\mathrm{O}}_{2\mathrm{m}\mathrm{a}\mathrm{x}}$ (%) than RA, with no differences in age. No significant age x training interactions were observed for any anthropometric variable or exercise characteristic.

### Echocardiography

#### Influence of ageing and exercise on LV structure

LV structure and volumes are presented in Table 2. No age or training effects were observed for IVS, PWT, or MWT. Similarly, scaled IVS, PWT, and MWT did not differ between age, while all were larger in trained than RA. LVEDD (absolute but not indexed) was smaller in older than younger cohorts, whereas absolute and indexed LVEDD were larger in trained than RA. RWT did not differ between age or training status. Absolute and indexed (height^2.7^ and BSA) LVM were not different between young and old, which were greater in trained than RA. Absolute LV length was smaller in older than younger cohorts, which demonstrated a trend towards being larger in trained than RA. When scaled to BSA, LV length did not differ based on age, but was larger in trained than RA. Concentricity, sphericity index, LV outflow tract cross-sectional area, and diameter were not different between ages or training statuses. Absolute LVEDV and LVESV were greater in trained than RA and smaller in older than younger cohorts. Similarly, LVEDV and LVESV scaled to BSA were greater in trained than RA, yet similar between ages. SV and SV index were larger in trained than RA, but did not differ between ages. $\dot{\mathrm{Q}}$ was lower in old than young cohorts and did not differ based on training status. In contrast, $\dot{\mathrm{Q}}$ index was greater in trained than RA, which was unremarkable between ages.

**Table 2:** Left-ventricular structure, geometry, and volumes in young recreationally active (Y_RA_), young trained (Y_T_), old recreationally active (O_RA_), and old trained (O_T_) participants

	Young		Old		*P* value		
	Recreationally active (Y_RA_)	Trained (Y_T_)	Recreationally active (O_RA_)	Trained (O_T_)	Age	Training	Interaction
IVS (mm)	9.7 ± 1.0	9.7 ± 1.0	9.6 ± 1.0	10.5 ± 1.1	0.29	0.18	0.21
IVS index [mm/(m^2^)^0.5^]	6.9 ± 0.7	7.0 ± 0.5	6.8 ± 0.7	7.8 ± 0.8	0.07	**0.02**	0.06
LVEDD (mm)	48.9 ± 2.0	51.6 ± 3.0	46.4 ± 2.7	49.6 ± 3.9	**0.03**	**0.004**	0.79
LVEDD index [mm/(m^2^)^0.5^]	34.7 ± 1.3	37.1 ± 1.5	31.6 ± 4.6	37.0 ± 2.1	0.08	** < 0.01**	0.10
PWT (mm)	10.1 ± 0.8	10.5 ± 1.4	9.7 ± 1.3	10.2 ± 1.0	0.32	0.24	0.90
PWT index [mm/(m^2^)^0.5^]	7.2 ± 0.5	7.5 ± 0.8	6.9 ± 1.0	7.6 ± 0.8	0.74	**0.04**	0.53
MWT (mm)	9.9 ± 0.6	10.1 ± 1.1	9.7 ± 0.9	10.3 ± 0.9	0.97	0.14	0.44
MWT [mm/(m^2^)^0.5^]	7.0 ± 0.4	7.3 ± 0.6	6.9 ± 0.7	7.7 ± 0.7	0.40	**0.01**	0.14
LVM (g)	177 ± 21	201 ± 44	157 ± 26	192 ± 37	0.20	**0.01**	0.60
LVM (g/m^2^)	89 ± 9	103 ± 16	80 ± 13	107 ± 17	0.59	** < 0.001**	0.16
LVM [g/(m^2^)^2.7^]	37 ± 4	41 ± 6	35 ± 6	44 ± 8	0.87	**0.01**	0.19
RWT	0.41 ± 0.02	0.39 ± 0.03	0.42 ± 0.05	0.42 ± 0.05	0.11	0.52	0.51
LV length (mm)	91 ± 5	96 ± 7	89 ± 5	91 ± 6	**0.05**	0.07	0.33
LV length [mm/(m^2^)^0.5^]	64 ± 3	69 ± 6	63 ± 3	68 ± 4	0.31	**0.002**	0.86
Concentricity [g/mL)^0.667^]	8.3 ± 1.2	7.5 ± 1.1	8.1 ± 1.5	7.9 ± 0.8	0.85	0.20	0.33
Sphericity index	1.9 ± 0.1	1.9 ± 0.2	1.9 ± 0.1	1.8 ± 0.2	0.81	0.35	0.31
LVOT diameter (mm)	21 ± 1	23 ± 2	21 ± 1	22 ± 2	0.25	0.09	0.42
LVEDV (mL)	98 ± 9	139 ± 25	85 ± 15	123 ± 20	**0.02**	** < 0.001**	0.82
LVEDV [mL/(m^2^)^1.5^]	35 ± 4	52 ± 9	32 ± 5	51 ± 6	0.30	** < 0.001**	0.58
LVESV (mL)	39 ± 2	58 ± 13	33 ± 9	47 ± 14	**0.02**	** < 0.001**	0.60
LVESV [mL/(m^2^)^1.5^]	14 ± 2	21 ± 4	12 ± 2	19 ± 5	0.09	** < 0.001**	0.99
LVOT CSA (cm^2^)	3.5 ± 0.5	4.0 ± 0.7	3.4 ± 0.4	3.6 ± 0.7	0.25	0.07	0.42
VTI (cm)	21.6 ± 4.6	25.1 ± 3.5	19.5 ± 2.6	24.8 ± 5.0	0.34	**0.002**	0.49
SV (mL)	75 ± 14	102 ± 24	67 ± 13	89 ± 21	0.10	** < 0.001**	0.69
SV index [mL/(m^2^)^1.5^]	27 ± 5	37 ± 7	24 ± 3	37 ± 9	0.47	** < 0.001**	0.59
$\dot{Q}$ (L min^−1^)	4.57 ± 1.25	4.77 ± 0.63	3.64 ± 0.85	4.12 ± 0.79	**0.009**	0.24	0.63
$\dot{Q}$ index (L min/(m^2^)^1.5^)	1.65 ± 0.50	1.79 ± 0.30	1.30 ± 0.20	1.71 ± 0.30	0.06	**0.01**	0.22

*CSA* cross-sectional area; *IVS* interventricular septum; *LVEDD* left-ventricular end-diastolic diameter; *PWT* posterior wall thickness; *MWT* mean wall thickness; *LVM* left-ventricular mass; *RWT* relative wall thickness; *LV* left ventricular; *LVOT* left-ventricular outflow tract; *VTI* velocity time integral; *LVEDV* left-ventricular end-diastolic volume; *LVESV* left-ventricular end-systolic volume; *SV* stroke volume; $\dot{Q}$ cardiac output

Data are means ± SD. Significant main effects are highlighted in bold

*P* ≤ 0.050. vs. Y_RA_; † vs. Y_T_; ‡ vs. O_RA_

#### Influence of ageing and exercise on conventional LV function

Conventional indices of functional systolic and diastolic dynamics are presented in Table 3. No differences based on ageing or training status were observed for EF or FS. In contrast, septal, lateral and averaged s’ were lower in older than younger cohorts, which were indistinguishable between trained and RA. However, differences based on ageing for average *s*′ were removed when scaled to BSA. Early diastolic function [E wave, septal, lateral, and averaged (absolute and indexed) *e*′] was lower in older than younger cohorts, while late diastolic filling [A wave, septal, lateral, and averaged (absolute and indexed) *a*′] was greater in older than younger cohorts. Together, *E*/*A*, *e*′/*a*′ were lower in older than younger, whereas estimated filling pressure (*E*/*e*′) was greater in the former. Lateral *e*′ was greater in trained than RA, with a significant interaction which revealed that Y_T_ had greater tissue velocity than the other groups. Average *e*′ (absolute and indexed) were greater in trained than RA without a significant interaction effect. Finally, *e*′/*a*′ was greater in trained than RA, yet the interaction effect identified that this was in the main due to being larger in Y_T_.

**Table 3:** Ultrasonic measures of conventional left-ventricular (LV) systolic and diastolic function in young recreationally active (Y_RA_), young trained (Y_T_), old recreationally active (O_RA_), and old trained (O_T_) participants

	Young		Old		*P* value		
	Recreationally active (Y_RA_)	Trained (Y_T_)	Recreationally active (O_RA_)	Trained (O_T_)	Age	Training	Interaction
Systolic function							
FS (%)	33 ± 3	30 ± 6	35 ± 3	33 ± 6	0.16	0.11	0.52
EF (%)	60 ± 4	59 ± 3	61 ± 6	61 ± 6	0.22	0.55	0.81
Septal *s*′ (cm/s)	9 ± 1	8 ± 1	8 ± 1	8 ± 1	**0.04**	0.71	0.94
Lateral *s*′ (cm/s)	10 ± 3	12 ± 3	9 ± 2	9 ± 3	**0.03**	0.36	0.57
Average *s*′ (cm/s)	10 ± 2	10 ± 2	9 ± 1	9 ± 2	**0.03**	0.52	0.52
Average *s*′ index [(cm/s)/cm]	1.04 ± 0.17	1.05 ± 0.21	0.95 ± 0.15	0.95 ± 0.21	0.11	0.94	0.94
Diastolic function							
E wave (cm/s)	73 ± 15	73 ± 11	61 ± 13	59 ± 14	**0.004**	0.72	0.83
A wave (cm/s)	40 ± 10	38 ± 7	56 ± 7	54 ± 12	** < 0.001**	0.62	0.96
*E*/*A*	1.98 ± 0.74	2.01 ± 0.56	1.13 ± 0.29	1.15 ± 0.42	** < 0.001**	0.89	0.98
Septal *e*′ (cm/s)	10 ± 3	12 ± 3	7 ± 1	8 ± 2	** < 0.001**	0.09	0.51
Lateral *e*′ (cm/s)	13 ± 4	17 ± 2*	9 ± 2*†	10 ± 2†	** < 0.001**	**0.001**	**0.04**
Septal *a*′ (cm/s)	8 ± 2	6 ± 1	9 ± 1	9 ± 1	** < 0.001**	0.09	0.09
Lateral *a*′ (cm/s)	7 ± 2	7 ± 2	9 ± 1	9 ± 1	** < 0.001**	0.42	0.42
Average *e*′ (cm/s)	12 ± 3	15 ± 2	8 ± 2	9 ± 2	** < 0.001**	**0.01**	0.10
Average *a*′ (cm/s)	8 ± 2	7 ± 2	10 ± 1	10 ± 1	** < 0.001**	0.12	0.12
Average *e*′ index [(cm/s)/cm]	1.27 ± 0.34	1.53 ± 0.15	0.89 ± 0.17	0.97 ± 0.20	** < 0.001**	**0.02**	0.20
Average *a*′ index [(cm/s)/cm]	0.85 ± 0.18	0.68 ± 0.22	1.05 ± 0.14	1.03 ± 0.10	** < 0.001**	0.09	0.13
Average *e*′/*a*′	1.58 ± 0.68	2.45 ± 0.64*	0.87 ± 0.23*†	0.95 ± 0.27*†	** < 0.001**	**0.01**	**0.02**
Average *E*/*e*′	6.66 ± 1.51	4.99 ± 0.78	7.81 ± 0.98	6.94 ± 1.31	**0.03**	0.10	0.13

*FS* fractional shortening; *EF* ejection fraction; *s*′ systolic tissue velocity; *E wave* early diastolic mitral inflow velocity; *A wave* late diastolic mitral inflow velocity; *E*/*A* ratio of early-to-late mitral inflow velocity; *e*′ early diastolic tissue velocity; *a*′ late diastolic tissue velocity; *e*′/*a*′ ratio of early-to-late diastolic tissue velocity; *E*/*e*′ ratio of early mitral inflow velocity-to-early diastolic tissue velocity. Significant findings are highlighted in bold.

Data are means ± SD. Significant main effects are highlighted in bold

*P* ≤ 0.050. * vs. Y_RA_; † vs. Y_T_; vs. O_RA_

### Influence of ageing and exercise on peak left-ventricular strain and strain rate

#### Longitudinal strain and strain rate

Layer-specific longitudinal strain and SR are presented in Table 4. Between-group comparisons of 4-chamber, 2-chamber, averaged longitudinal strain, and strain gradient were unremarkable. Older had lower apical 4-chamber SR_S_ than younger cohorts, which was also lower in trained than RA. Whereas 2-chamber and averaged SR_S_ were lower in trained than RA, but did not differ between ages. Four-chamber, 2-chamber, and averaged SR_E_ were lower, with higher SR_A_ in older than younger cohorts. No training effect was noted for SR_E_ or SR_A_ from 4-chamber; however, 2-chamber and averaged SR_A_ were lower in trained than RA.

**Table 4:** Peak layer-specific longitudinal strain and strain rate in young recreationally active (Y_RA_), young trained (Y_T_), old recreationally active (O_RA_) and old trained (O_T_) participants

	Young		Old		*P* value		
	Recreationally active (Y_RA_)	Trained (Y_T_)	Recreationally active (O_RA_)	Trained (O_T_)	Age	Training	Interaction
Apical 4-chamber							
Endocardium (%)	− 22.0 ± 3.0	− 20.8 ± 1.4	− 21.1 ± 2.1	− 21.7 ± 2.9	0.99	0.67	0.25
Myocardium (%)	− 19.9 ± 2.4	− 18.5 ± 1.3	− 19.2 ± 1.8	− 19.2 ± 2.6	0.95	0.31	0.32
Epicardium (%)	− 17.9 ± 2.1	− 16.4 ± 1.3	− 17.5 ± 1.5	− 17.0 ± 2.4	0.83	0.12	0.45
Layer gradient	4.2 ± 1.5	4.4 ± 0.5	3.6 ± 0.8	4.7 ± 1.3	0.69	0.07	0.22
SR_S_ (s^−1^)	− 1.05 ± 0.14	-0.94 ± 0.06	− 0.94 ± 0.09	− 0.89 ± 0.09	**0.01**	**0.01**	0.30
SR_E_ (s^−1^)	1.45 ± 0.44	1.50 ± 0.18	0.92 ± 0.22	1.05 ± 0.34	** < 0.001**	0.37	0.71
SR_A_ (s^−1^)	0.70 ± 0.19	0.53 ± 0.18	0.86 ± 0.21	0.90 ± 0.17	** < 0.001**	0.27	0.09
Apical 2-chamber							
Endocardium (%)	− 22.0 ± 3.0	− 23.2 ± 1.4	− 23.5 ± 3.3	− 21.5 ± 4.1	0.95	0.70	0.14
Myocardium (%)	− 20.0 ± 2.4	− 20.7 ± 1.2	− 21.0 ± 2.9	− 19.3 ± 3.7	0.80	0.56	0.18
Epicardium (%)	− 18.2 ± 2.0	− 18.4 ± 1.1	− 18.8 ± 2.7	− 17.2 ± 3.4	0.65	0.38	0.27
Layer gradient	3.8 ± 1.5	4.7 ± 1.0	4.7 ± 1.8	4.4 ± 1.0	0.49	0.48	0.14
SR_S_ (s^−1^)	− 1.03 ± 0.13	− 0.97 ± 0.10	− 1.04 ± 0.14	− 0.90 ± 0.19	0.46	**0.04**	0.43
SR_E_ (s^−1^)	1.39 ± 0.25	1.62 ± 0.20	1.04 ± 0.23*†	0.90 ± 0.29*†	** < 0.001**	0.55	**0.03**
SR_A_ (s^−1^)	0.70 ± 0.16	0.55 ± 0.10	1.01 ± 0.24*†	0.87 ± 0.16†	** < 0.001**	**0.01**	0.93
Apical 4- and 2-chamber average							
Endocardium (%)	− 22.0 ± 2.7	− 22.0 ± 1.1	− 22.5 ± 2.2	− 21.8 ± 3.3	0.85	0.63	0.66
Myocardium (%)	− 19.9 ± 2.2	− 19.6 ± 1.0	− 20.3 ± 1.8	− 19.4 ± 2.9	0.91	0.38	0.68
Epicardium (%)	− 18.0 ± 1.9	− 17.4 ± 0.9	− 18.3 ± 1.6	− 17.3 ± 2.6	0.96	0.18	0.73
Layer gradient	4.0 ± 1.4	4.5 ± 0.5	4.2 ± 1.2	4.5 ± 1.2	0.74	0.23	0.68
SR_S_ (s^−1^)	− 1.05 ± 0.12	− 0.96 ± 0.07	− 0.99 ± 0.10	− 0.90 ± 0.12	0.11	**0.01**	0.90
SR_E_ (s^−1^)	1.42 ± 0.32	1.56 ± 0.13	0.97 ± 0.18	1.00 ± 0.27	** < 0.001**	0.27	0.51
SR_A_ (s^−1^)	0.70 ± 0.17	0.51 ± 0.17	0.97 ± 0.14	0.90 ± 0.15	** < 0.001**	**0.02**	0.33

*SR*_*S*_ strain rate systole; *SR*_*E*_ strain rate early diastole; *SR*_*A*_ strain rate late diastole

Data are means ± SD. Significant main effects are highlighted in bold

*P* ≤ 0.050. * vs. Y_RA_; † vs. Y_T_; vs. O_RA_

#### Circumferential and radial strain and strain rate

Layer-specific circumferential strain, SR, and radial strain and SR are presented in Table 5. Between-group comparisons of layer-specific basal, apical and averaged circumferential strain and radial strain were largely unremarkable. However, epicardial circumferential strain at the base was lower in old than young cohorts, which in turn contributed towards larger strain gradient and identified a higher basal strain gradient in O_T_ than Y_T_. Basal SR_S_ was lower in older than younger cohorts but not at the apex of when average. No differences between trained and RA were observed for SR_S._ Basal, apical, and global circumferential SR_E_ were lower, whereas SR_A_ were higher in older than younger cohorts. Diastolic SR were not different between trained and RA. Similarly, radial SR_A_ were higher in old than young, without difference based on training status. Radial SR_E_ did not differ based on age or training, while trained had greater global radial SR_S_ than RA without further differences noted.

**Table 5:** Peak layer-specific circumferential and radial strain and strain rate in young recreationally active (Y_RA_), young trained (Y_T_), old recreationally active (O_RA_) and old trained (O_T_) participants

	Young		Old		*P* value		
	Recreationally active (Y_RA_)	Trained (Y_T_)	Recreationally active (O_RA_)	Trained (O_T_)	Age	Training	Interaction
Circumferential–base							
Endocardium (%)	− 24.5 ± 4.7	− 23.3 ± 4.7	− 21.5 ± 6.6	− 27.8 ± 5.6	0.66	0.16	**0.04**
Myocardium (%)	− 16.2 ± 3.3	− 16.3 ± 4.0	− 13.2 ± 4.6	− 17.2 ± 4.0	0.41	0.12	0.13
Epicardium (%)	− 10.2 ± 3.0	− 11.1 ± 3.8	− 7.4 ± 3.5	− 9.5 ± 3.4	**0.05**	0.18	0.60
Layer gradient	14.3 ± 3.9	12.2 ± 2.9	14.2 ± 4.5	18.3 ± 4.3 †	**0.02**	0.43	**0.02**
SR_S_ (s^−1^)	− 0.99 ± 0.14	− 1.02 ± 0.20	− 0.75 ± 0.20	− 0.95 ± 0.21	**0.02**	0.07	0.17
SR_E_ (s^−1^)	1.35 ± 0.43	1.25 ± 0.33	0.94 ± 0.28	1.18 ± 0.30	**0.03**	0.49	0.12
SR_A_ (s^−1^)	0.47 ± 0.32	0.32 ± 0.16	0.57 ± 0.14	0.71 ± 0.26	**0.002**	0.99	0.05
Circumferential–apex							
Endocardium (%)	− 38.4 ± 8.4	− 37.8 ± 6.7	− 42.9 ± 6.3	− 40.4 ± 6.9	0.13	0.49	0.69
Myocardium (%)	− 26.3 ± 6.4	− 25.7 ± 5.4	− 26.4 ± 5.7	− 27.9 ± 5.9	0.53	0.81	0.59
Epicardium (%)	− 17.5 ± 6.3	− 18.1 ± 4.8	− 17.7 ± 5.9	− 20.2 ± 6.5	0.53	0.42	0.63
Layer gradient	− 21.0 ± 6.7	− 19.7 ± 5.4	− 25.1 ± 6.8	− 20.2 ± 6.9	0.27	0.14	0.38
SR_S_ (s^−1^)	− 1.67 ± 0.36	− 1.42 ± 0.17	− 1.50 ± 0.49	− 1.48 ± 0.31	0.60	0.23	0.31
SR_E_ (s^−1^)	2.39 ± 1.00	2.42 ± 0.70	1.81 ± 0.49	1.95 ± 0.71	**0.03**	0.73	0.81
SR_A_ (s^−1^)	0.86 ± 0.28	0.75 ± 0.20	1.26 ± 0.42	1.15 ± 0.23	** < 0.001**	0.22	0.99
Circumferential–global							
Endocardium (%)	− 31.5 ± 4.2	− 30.5 ± 4.2	− 32.2 ± 5.3	− 34.1 ± 5.7	0.18	0.77	0.37
Myocardium (%)	− 21.3 ± 3.4	− 21.0 ± 3.5	− 19.8 ± 4.5	− 22.5 ± 4.8	0.96	0.35	0.26
Epicardium (%)	− 13.8 ± 3.8	− 14.6 ± 3.0	− 12.6 ± 4.1	− 14.8 ± 4.4	0.68	0.23	0.54
Gradient	17.7 ± 2.9	15.9 ± 2.6	19.7 ± 4.2	19.3 ± 3.8	**0.02**	0.34	0.54
SR_S_ (s^−1^)	− 1.33 ± 0.21	− 1.22 ± 0.15	− 1.13 ± 0.31	− 1.22 ± 0.21	0.15	0.87	0.17
SR_E_ (s^−1^)	1.87 ± 0.51	1.84 ± 0.40	1.37 ± 0.31	1.56 ± 0.40	**0.01**	0.55	0.39
SR_A_ (s^−1^)	0.66 ± 0.27	0.54 ± 0.16	0.91 ± 0.25†	0.93 ± 0.14	** < 0.001**	0.41	0.28
Radial–base							
Strain (%)	38.9 ± 11.5	34.9 ± 10.6	34.6 ± 16.2	46.0 ± 11.6	0.40	0.36	0.06
SR_S_ (s^−1^)	2.75 ± 0.83	2.36 ± 1.07	2.77 ± 0.59	2.42 ± 1.13	0.89	0.22	0.94
SR_E_ (s ^−1^)	− 2.27 ± 0.60	− 1.99 ± 0.81	− 2.45 ± 0.87	− 2.09 ± 1.20	0.61	0.28	0.89
SR_A_ (s^−1^)	− 1.67 ± 0.50	− 0.79 ± 0.74	− 1.99 ± 0.94†	− 2.51 ± 1.12†	** < 0.001**	0.52	**0.02**
Radial–apex							
Strain (%)	32.4 ± 20.9	24.6 ± 16.2	24.7 ± 16.2	22.6 ± 11.9	0.39	0.37	0.60
SR_S_ (s^−1^)	2.04 ± 0.89	1.43 ± 0.46	1.82 ± 0.93	1.55 ± 0.74	0.85	0.10	0.51
SR_E_ (s^−1^)	− 2.65 ± 0.65	− 2.98 ± 1.56	− 2.49 ± 1.21	− 1.83 ± 0.76	0.09	0.67	0.19
SR_A_ (s^−1^)	− 0.52 ± 0.35	− 0.33 ± 0.30	− 1.07 ± 0.91	− 0.71 ± 0.59	**0.03**	0.19	0.69
Radial–global							
Strain (%)	34.6 ± 10.7	30.9 ± 8.9	29.7 ± 9.9	34.2 ± 7.8	0.79	0.90	0.19
SR_S_ (s^−1^)	2.38 ± 0.55	1.77 ± 0.26	2.29 ± 0.52	2.02 ± 0.76	0.66	**0.02**	0.37
SR_E_ (s^−1^)	− 2.45 ± 0.52	− 2.36 ± 0.77	− 2.47 ± 0.80	− 2.02 ± 0.84	0.52	0.29	0.46
SR_A_ (s^−1^)	− 1.09 ± 0.36	− 0.58 ± 0.35	− 1.53 ± 0.54	− 1.47 ± 0.65	** < 0.001**	0.09	0.19

*SR*_*S*_ strain rate systole; *SR*_*E*_ strain rate early diastole; *SR*_*A*_, strain rate late diastole

Data are means ± SD. Significant main effects are highlighted in bold

*P* ≤ 0.050. vs. Y_RA_; † vs. Y_T_; vs. O_RA_

### Left-ventricular strain and strain rate across the cardiac cycle

#### Longitudinal strain and strain rate

**Fig. 1:**
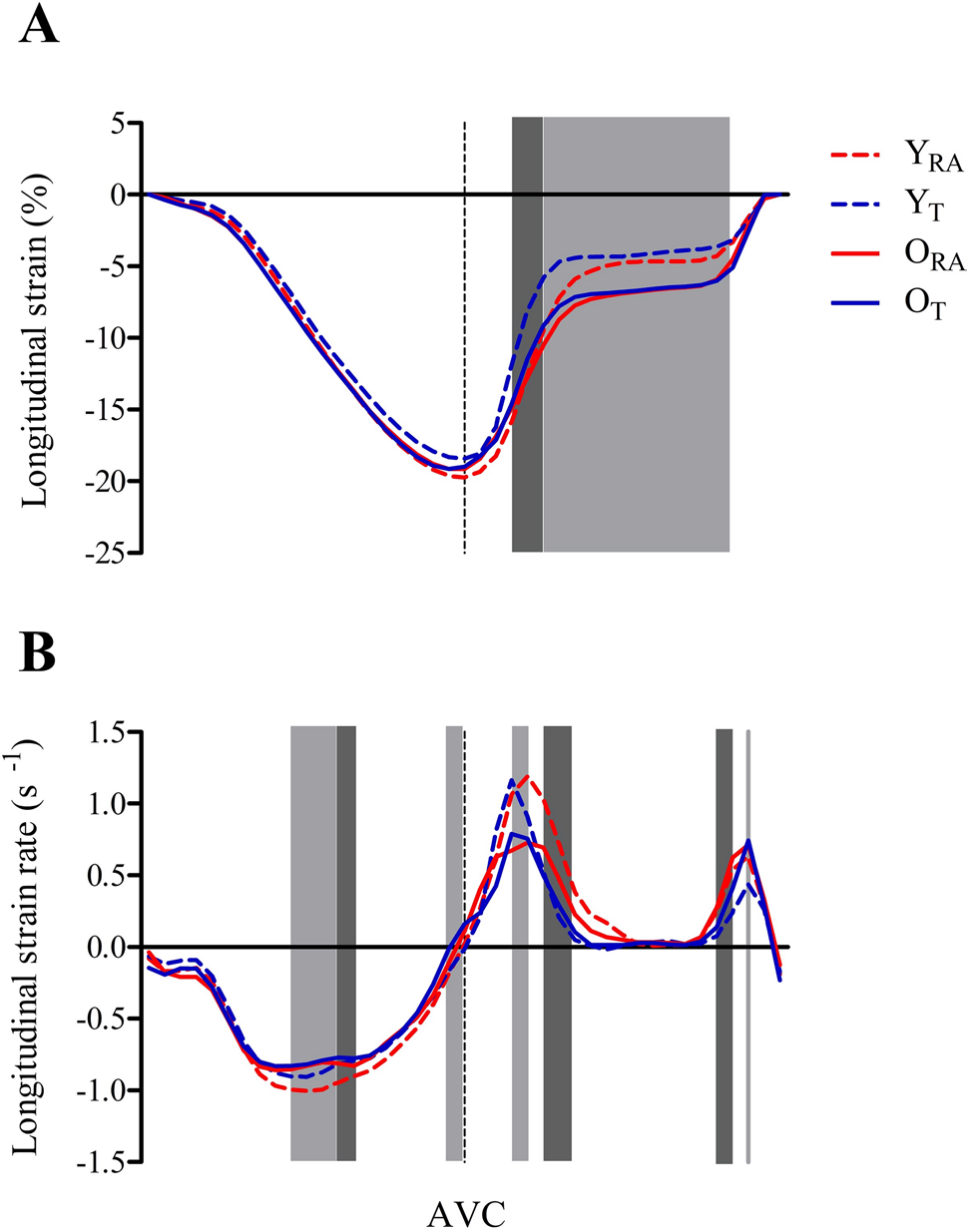
Longitudinal strain (**a**) and strain rate (**b**) across the cardiac cycle in 5% increments of systole and diastole for young recreationally active (Y_RA_), young trained (Y_T_), old recreationally active (O_RA_), and old trained (O_T_). Light shaded areas indicate a statistically significant main effect of age and dark shaded areas signify a training effect, both at *P* ≤ 0.05. AVC, aortic valve closure (end-systole [100%]). Data are presented as group means with error bars omitted for clarity

Longitudinal strain and SR throughout the cardiac cycle are illustrated in Fig. 1. During systole, between-group comparisons of 4-chamber longitudinal strain were unremarkable. In contrast, trained cohorts had lower strain (less negative) than RA 15–25% diastole. Moreover, older cohorts had higher strain (more negative) than younger cohorts between 25–85% (i.e., less lengthening), regardless of training status from 30%-85% diastole. Four-chamber longitudinal SR was lower from 45–60% and 95% systole in older than younger cohorts, yet was greater in the former than the latter at 100% systole. At 60–65% systole, trained had lower SR than RA. During diastole, SR was lower in older than young cohorts at 15–20%, yet higher in the former at 90%. Trained cohorts had lower SR at 25–35% and 80–85% than RA.

#### Circumferential strain and strain rate

**Fig. 2:**
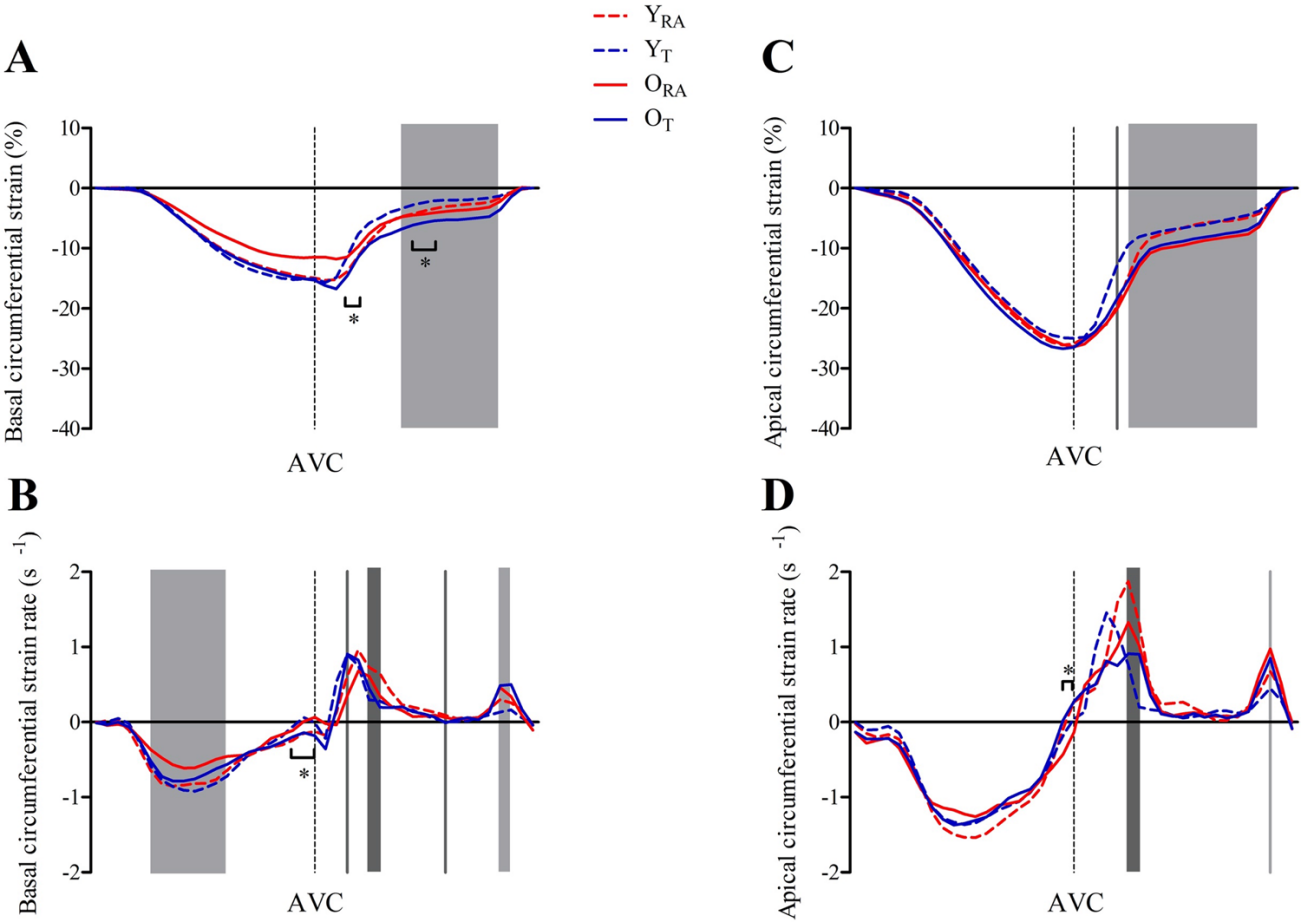
Circumferential strain [base (**a**); apex (**c**)] and strain rate [base (**b**); apex (**d**)] across the cardiac cycle in 5% increments of systole and diastole for young recreationally active (Y_RA_), young trained (Y_T_), old recreationally active (O_RA_), and old trained (O_T_). Light shaded areas indicate a statistically significant main effect of age and dark shaded areas signify a training effect, both at *P* ≤ 0.05. * represents a significant interaction effect with post hoc comparisons detailed in text. AVC, aortic valve closure (end-systole [100%]). Data are presented as group means with error bars omitted for clarity

Basal and apical circumferential strain and SR are illustrated in Fig. 2. During systole, between-group comparisons of basal circumferential strain were unremarkable. During diastole, an interaction effect was observed at 15–20% diastole, yet post hoc analysis did not identify between-group differences. Similarly, an interaction effect was observed at 45–55% diastole, with post hoc analysis identifying lower strain in Y_T_ than O_T_. Older cohorts had higher strain (more negative) than younger cohorts between 40 and 85% (i.e., less expansion), regardless of training status. Between-group comparisons of apical circumferential strain during systole were unremarkable. In contrast, at 20% diastole, strain was lower in trained than RA. Moreover, between 25 and 85% diastole, older cohorts had more strain (i.e., more negative) than younger cohorts (i.e., less expansion), regardless of training status.

Basal circumferential SR was lower in older than young cohorts from 25–60% systole. An interaction effect was observed at 90–100% systole; however, post hoc analysis did not identify significant between-group differences. During diastole, older cohorts had higher SR than young at 85–90%. Trained cohorts had higher SR than RA at 15% diastole, yet lower SR than RA at 25–30% and 60% diastole.

A significant interaction effect was observed for apical circumferential SR at 95–100% systole. Post hoc analysis identified lower SR in O_T_ than O_RA_ at 95%, yet not between-group differences at 100%. At 25–30% diastole, trained had lower SR than RA. Older cohorts had higher SR at 90% diastole than younger cohorts.

## Discussion

The main findings from this study were: (1) diastolic lengthening (longitudinal) and expansion (apical and basal circumferential) were lower during mid-diastole in older than younger groups. (2) The age-associated reductions in mid-diastolic lengthening and expansion were not attenuated in chronically trained older exercisers. (3) There were only minor differences in peak systolic longitudinal and circumferential strain, within myocardial layers according to healthy ageing or training status. Thus, the influence of ageing and exercise on LV longitudinal and circumferential strain is not synonymous across the entire cardiac cycle, but appears to be dependent on systole and diastole. Importantly, using non-invasive STE derived techniques, this study presents novel characterization of strain mechanics and, subsequently, plasticity in both young and old RA and athletic hearts of men.

### Left-ventricular strain–diastole

#### Influence of healthy ageing

Less lengthening (longitudinal) and expansion (apical and basal circumferential) were evident in the older than younger cohorts during mid-diastole (diastasis). We speculate that without temporally aligned LV pressure, these differences are a consequence of compromised compliance (increased stiffness and reduced distension) during diastasis with advancing age. Similar modifications are known to occur within the senescent human LV, including increased end-diastolic stiffness with reduced chamber compliance and distensibility through multiple reports of invasive pressure–volume curves (Arbab-Zadeh et al. 2004; Fujimoto et al. 2012). While related, our use of strain characterizes LV plasticity throughout diastole, while the aforementioned studies assess the pressure–volume relationship at end-diastole and, thus, the physiological interpretations between methodologies differ slightly.

Lower lengthening and expansion during diastasis (i.e., mid-diastolic plateau in strain) with ageing may reflect a stiffer LV following the active period of relaxation, yet prior to atrial systole. In turn, and in accordance with the strain profiles, the LV does not return to its baseline length until after atrial systole, which may represent a greater reliance on atrial systole to return LV fibers to their original length. Indeed, the atrial contribution to ventricular lengthening in strain may need to increase to normalize LVEDV, which is evidenced by faster A wave and SR_A_ with ageing.

The implications of lower mid-diastolic lengthening and expansion in older cohorts at this moment are unclear; however, it would not appear to impair LV filling, since difference in LVEDV index was unremarkable between RA groups. Yet, further interpretation would be overly speculative due to the sample sizes in this study. Moreover, whether the reduced lengthening and expansion in the older cohorts represent altered intrinsic myocardial properties or elastic modifications of extracellular structures (Smiseth 2018) cannot be fully determined from this study. Without additional level-specific (base-apex gradient) analysis of longitudinal strain, it is difficult to ascertain whether the lower longitudinal lengthening in older age is uniform along with the LV or specific to the apical and/or basal regions. Indeed, a region-specific pattern of diastolic deformation is plausible, since longitudinal systolic strain has demonstrated subtle increases and decreases in apical and basal strain, respectively with advancing age (Abou et al. 2017). Thus, regional dissemination of strain during diastole warrants further work that specifically examines whether altered diastolic strain profiles with ageing are localized or uniform along with the LV.

#### Influence of exercise training on age-associated differences

An important and novel finding from this study was that the lower LV lengthening and expansion with ageing was not attenuated in chronically trained exercisers and, consequently, ageing rather than training status appears to be dominant in altering mid-diastolic strain profiles. The ramifications of this in O_T_ are unclear, yet the lack of compensation with chronic exercise training does not seem to impair maximum diastolic filling. Although, these observations were made at rest and, thus, the functional reserve during physiological stress, such as exercise, for example, may provide further insights into the functionality between trained and RA older cohorts. Indeed, it would be of further interest to investigate the response of these novel measures from rest to exercise, up to the merging of early and late diastolic filling.

Despite the reduced LV lengthening, O_T_ had larger LVEDV than both Y_RA_ and O_RA_; therefore, alterations in diastolic strain and LVEDV may not be interdependent. Indeed, both trained groups had similar diastolic strain profiles but larger LVEDV than their age-matched counterparts. Larger LVEDV was evident in trained groups without heightened reliance on atrial systole (A wave, SR_A_) compared to their RA counterparts, suggesting that it is unlikely that atrial systole is responsible for their larger volumes. Indeed, trained groups did show greater averaged e’ than RA, which may have improved early diastolic filling. Also, trained groups had lower HR than RA which could have lengthened the filling time and, in turn, facilitated a larger LVEDV. Moreover, it is difficult to determine whether larger EDV in trained groups represents augmented end-diastolic distensibility, as has previously been suggested at similar filling pressures (PCWP) with increasing chronic exercise training doses (Bhella et al. 2014). More likely, however, is physiological adaptation of an enlarged chamber size (EDD index and LV length index), which may account for larger LVEDV in trained groups. Still, it is unclear whether a combination of remodeling and superior end-diastolic distension could be responsible.

Findings from this study contrast with a previous study reporting an age-associated reduction in LV compliance was prevented entirely in master athletes (Arbab-Zadeh et al. 2004). The reasons for the contrasting findings are not entirely clear, at least in consideration of participant characteristics. Our O_T_ participants began training at ~ 31 ± 11 years of age, which had done so ≥ 9 years at a frequency of 4–5 sessions per week. Moreover, we purposely selected to include only those who started exercise training before 64 years of age, since prior work demonstrated that training in over 65 years old did not alter LV compliance (Fujimoto et al. 2012). Together, these characteristics are within the constraints of other work having reported benefits of chronic exercise on LV compliance and stiffness (Matelot et al. 2016; Howden et al. 2018). Although, lifelong exercise has been documented previously as 25 years; whether this duration is required as a minimum to preserve compliance warrants clarification (Bhella et al. 2014). Alternatively, although training duration and exposure were quantified, there may be underlying relationships between the intensity of training necessary to prevent age-associated differences in LV strain profiles, and therefore, this factor should be investigated further. Moreover, while we draw comparisons with existing work, it is worth noting that dissimilar findings may relate to the differing methods of assessment. Although compliance and stiffness (Matelot et al. 2016; Howden et al. 2018), and strain (as used in this study) are related, they are derived using a variety of techniques and correspondingly also have different physiological interpretations.

### Left-ventricular strain–systole

Longitudinal strain was similar between young and old cohorts within all myocardial layers and throughout the systolic period. Comparable peak strain profiles agree with those reporting that longitudinal is not altered with ageing (Yingchoncharoen et al. 2013; Kocabay et al. 2014; Nagata et al. 2017). Similarly, the longitudinal transmural gradient was not altered by healthy ageing, which concurs with the previous reports (Nagata et al. 2017; Alcidi et al. 2018). Conversely, the present observations disagree with others who report an age-associated decrease in longitudinal strain (Dalen et al. 2010; Sun et al. 2013; Hung et al. 2017; Alcidi et al. 2018) and it remains unclear why this inconsistency exists within the literature.

We purposely employed a stringent set of exclusion criteria to isolate healthy ageing from other confounding factors such as cardiovascular diseases. This approach facilitated our ability to investigate ‘true’ ageing (i.e., differences in the absence of overt disease) and its interaction with exercise training independently. In turn, the healthy status of our participants could provide some explanation for the maintained strain with advancing age. Indeed, reduced longitudinal strain is indicative of early LV dysfunction and commonly observed in pathological conditions and hypertensive populations (Galderisi et al. 2010; Butz et al. 2011).

Circumferential strain within the endocardial or myocardial layers did not differ between young and old, which agrees with other work (Zghal et al. 2011; Yingchoncharoen et al. 2013; Kocabay et al. 2014; Nagata et al. 2017). Minor differences in basal epicardial strain and strain gradient were observed between young and old; however, the interaction effect in the latter identified that this was largely due to O_T_. Although the strain gradient at the apex remained stable with advancing age, a larger global circumferential strain gradient was observed in older than younger groups, which disagrees with others (Nagata et al. 2017). Based on the current study, it remains inconclusive whether this is the consequence of altered endocardial or epicardial strain, or a small change in both directions.

Comparable longitudinal strain between trained and RA cohorts supports the previous meta-analyses in young and older age (Beaumont et al. 2016, 2018). Compensatory increases with chronic exercise may not be necessary if reduced strain is not part of the healthy ageing process. This may explain the disparate findings with recent work identifying preserved longitudinal strain in lifelong exercisers, compared to the age-associated decline with sedentary ageing (Howden et al. 2017). Study findings are heterogeneous, however, and may be related to variations in LV geometry and/or diastolic volumes (Oxborough et al. 2016; Forsythe et al. 2018a). After preload adjustments and comparisons made at similar LVEDV index, longitudinal strain was lower in trained seniors than sedentary young and seniors, highlighting the preload dependency of longitudinal strain in exercise and ageing (Howden et al. 2017). In this study, while LVEDV was larger in trained groups, strain was comparable and, thus, adaptations to LVEDV and strain are not interdependent.

### Conventional measures of cardiac structure and function

Trained groups demonstrated known physiological adaption to exercise training, including greater EDD, LVEDV, SV, and LVM compared with RA groups. In particular, similar differences in mean LVMi were observed in the present study (27 g m^2^) and previously meta-analysis (28 g m^2^) (Beaumont et al. 2018).

Comparable resting systolic function, clearly differentiated age-associated decline in global diastolic function, that is not attenuated with chronic exercise concurs with the previous work (Molmen et al. 2012; Carrick-Ranson et al. 2012; Olsen et al. 2015). In contrast to Beaumont et al. (2018), O_T_ did not demonstrate superior trans-mitral function than their age-matched untrained counterparts. In O_RA_, however, the ability to be relatively inactive for many years into old age and still avoid lifestyle-associated diseases known to occur in sedentary populations means that genetic factors cannot be neglected. Although, some O_RA_ were involved with recreational exercise, including hill walking, gym-based exercises, and walking soccer for example, which may have reduced the physiological margin. Therefore, the health status of untrained older men may warrant further study to explore biological from chronological ageing.

## Limitations

The present study is not without limitations. Our definition of RA does not preclude some (albeit minimal) engagement with exercise, which may act as a confounder. However, these sports and the participants’ involved were intermittent and the limitations of classifying team-based sports as highly dynamic given differences in playing position have been acknowledged (Mitchell et al. 2005). Others have adopted a similar approach by including individuals involved with low levels of continuous exercise despite some participation in team sports (Carrick-Ranson et al. 2012). Aerobically trained males of various disciplines were recruited, including runners, cyclists, and triathletes which represent differing static loading with a high dynamic component (Mitchell et al. 2005). Although meta-analysis has previously suggested that training type may influence STE derived LV mechanics (Beaumont et al. 2016), other work specifically investigating runners (high dynamic, low static) and rowers (high dynamic, high static) reported similar longitudinal and circumferential strain between groups (Wasfy et al. 2015). Second, training frequencies, volumes, and histories were recorded through self-reported questionnaire, and thus, recall error is possible which may have resulted in activity levels being overestimated or underestimated. While the present study has identified novel LV characterization of strain, these related to rest only, and thus, future work should explore the CV reserve and plasticity upon physiological exercise stress.

Participants were enrolled from the general population after advertising for healthy individuals without cardiovascular diseases. Of the males recruited, 18% (12/68) of older men were still excluded because of medications or medical conditions. Whilst exclusion of such conditions was intentional and limits generalizability, the primary aim of the study was to understand the cardio-physiology of exercise during ageing. Consequently, our use of ‘healthy ageing’ differs from the epidemiological definition by the World Health Organization (World Health Organisation 2015). Accordingly, before the present findings can be generalized to the wider community, further investigation is warranted in the general population irrespective of the presence of cardiovascular diseases. Moreover, as a consequence of the stringent inclusion criteria used in this study, the sample sizes were small and, thus, replication work is required to confirm the present findings. Finally, only men were included for the present study, and based on sex-dependent LV mechanics with ageing (Kocabay et al. 2014; Hung et al. 2017), findings should not be generalized to the female population. There exists a paucity of data in the female community and should, therefore, be investigated in relation to ageing and exercise.

## Conclusion

Mid-diastolic longitudinal lengthening and circumferential expansion were lower in older than younger RA males, which were not mitigated in chronically trained older adults. In the main, systolic strain did not differ between young and old groups, irrespective of exercise training status. Together, the influence of ageing and exercise on LV longitudinal and circumferential strain is not synonymous across the entire cardiac cycle, but appears to be dependent on systole and diastole. Through non-invasive STE-derived techniques, this study has documented novel insights into strain mechanics and LV plasticity in young and old RA and athletic hearts of men.

## Electronic supplementary material

Below is the link to the electronic supplementary material.

Supplementary file1 (DOCX 14 kb)

Supplementary file2 (DOCX 422 kb)

Supplementary file3 (DOCX 742 kb)

## Abbreviations

BMI: Body mass index
BSA: Body surface area
ECG: Electrocardiography
HR: Heart rate
LV: Left ventricle
LVEDD: Left-ventricular end-diastolic diameter
LVEDV: Left-ventricular end-diastolic volume
LVMi: Left-ventricular Mass Index
SR_A_: Late diastolic strain rate
SR_E_: Early diastolic strain rate
SR_S_: Systolic strain rate
STE: Speckle tracking echocardiography
SV: Stroke volume
$\dot{V}0_{{2\max }}$: Maximal oxygen consumption
