# Supplementary material for: Long-term athletic training does not alter age-associated reductions of left-ventricular mid-diastolic lengthening or expansion at rest
Source: Eur J Appl Physiol. 2020 Jul 4;120(9):2059–73. doi: 10.1007/s00421-020-04418-1 (PMC7419356; doi:10.1007/s00421-020-04418-1)
Supplement: Supplementary file 1 — Supplementary file1 (DOCX 14 kb) [file 421_2020_4418_MOESM1_ESM.docx]

**Supplementary material 1 - Training characteristics of trained populations**

YT participants had been training for at least 1.5 years and at the time of assessment reported consistent training habits of 3-7 days per week, ≥ 3.5 hours per week (3.5 – 17 hours) for ≥ 2 months (2 months – 12.5 years). Those involved with running did an average mileage of 26 ± 18 miles per week and those engaged with cycling, rode 134 ± 111 miles per week.

The OT group consisted of distance runners (>5 km events), cyclists and triathletes. OT had been engaged with exercise for at least 10 years and at the time of assessment reported a training consistently of 4-7 days per week, ≥ 5 hours per week (5 – 16 hours) for ≥ 9 years (9 – 49 years), without >1-year absence of training. Those involved with running, ran 39 ± 14 miles per week and those who performed cycling, rode 130 ± 136 miles per week. Also, those who swam performed a weekly distance of 2 ± 2 miles per week.
