# Supplementary material for: Long-term athletic training does not alter age-associated reductions of left-ventricular mid-diastolic lengthening or expansion at rest
Source: Eur J Appl Physiol. 2020 Jul 4;120(9):2059–73. doi: 10.1007/s00421-020-04418-1 (PMC7419356; doi:10.1007/s00421-020-04418-1)
Supplement: Supplementary file 2 — Supplementary file2 (DOCX 422 kb) [file 421_2020_4418_MOESM2_ESM.docx]

**Supplementary material 2 – Example images**


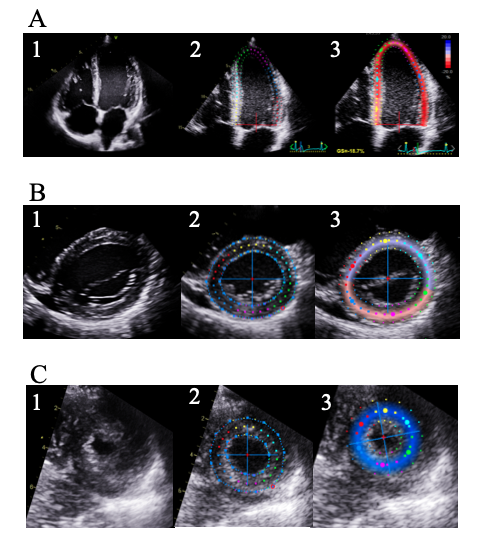


Figure 1. Representation of LV two-dimensional speckle tracking echocardiography for longitudinal strain – apical 4-chamber (A), basal mechanics – mitral valve short-axis level (B) and apical mechanics – apex short-axis (C). An appropriate image is chosen (1) before a region of interest (ROI) is placed around the entire myocardium, encompassing the endocardial and epicardial borders (2). After verification of successful tracking by vendor and operator, strain, strain rate and rotations are determined frame-by-frame (3) before being exported for offline analysis.
