# Supplementary material for: Long-term athletic training does not alter age-associated reductions of left-ventricular mid-diastolic lengthening or expansion at rest
Source: Eur J Appl Physiol. 2020 Jul 4;120(9):2059–73. doi: 10.1007/s00421-020-04418-1 (PMC7419356; doi:10.1007/s00421-020-04418-1)
Supplement: Supplementary file 3 — Supplementary file3 (DOCX 742 kb) [file 421_2020_4418_MOESM3_ESM.docx]

**Supplementary material 3 - STE reproducibility of LV strain at peak and across the cardiac cycle**

Eight males (27 ± 3 years) were used to assess the reproducibility of STE derived LV mechanics. Individuals were asked to refrain from alcohol, caffeine and exercise (24 hours) prior to the assessment. After an initial supine rest period of 10 minutes, two scans were conducted that were separated by approximately 20 minutes to minimise the potential biological variation. Participants remained supine throughout this period between scans. Methodological procedures were replicated between scans and conducted as outlined in chapter 4.4. Similar to a previous protocol reporting the reproducibility of echocardiographic parameters, the sonographer was blinded to the images acquired during scan 1 (Oxborough, George and Birch, 2012), with images captured at the same frame rate between scans. For the purpose of reproducibility, peak LV strains are representative of mid-wall shortening (longitudinal [apical 4-chamber] and circumferential [basal, apical and global]) and thickening (radial [basal, apical and global]).

Coefficient of variation (CoV) was calculated for each individual between scan 1 and scan 2 and then averaged, using the equation: CoV = $\frac{Standard deviation}{\mathrm{Mean}} x 100.$ Intra-observer test-retest reproducibility for LV strain and strain rate are presented in Table 1 and twist mechanics in Table 2. Mean temporal LV mechanics from test 1 and test 2 are illustrated in Figures 1, 2 and 3 for the main variables displayed in the following chapters within this thesis.

Table 1. Reproducibility of LV strain and strain rate.

|  | Scan 1 | Scan 2 | CoV (%) |
| --- | --- | --- | --- |
| *Longitudinal – Apical 4-chamber* |  |  |  |
| Strain (%) | -18.1 ± 1.9 | -17.8 ± 1.9 | 2.9 |
| SR_S_ (s ^-1^) | -0.95 ± 0.13 | -0.92 ± 0.14 | 3.5 |
| SR_E_ (s ^-1^) | 1.45 ± 0.20 | 1.33 ± 0.17 | 10.4 |
| SR_A_ (s ^-1^) | 0.56 ± 0.21 | 0.52 ± 0.15 | 13.9 |
| *Circumferential - Basal* |  |  |  |
| Strain (%) | -14.5 ± 2.6 | -15.5 ± 3.3 | 10.8 |
| SR_S_ (s ^-1^) | -0.91 ± 0.11 | -0.88 ± 0.11 | 8.4 |
| SR_E_ (s ^-1^) | 1.13 ± 0.36 | 1.18 ± 0.39 | 21.2 |
| SR_A_ (s ^-1^) | 0.35 ± 0.16 | 0.40 ± 0.15 | 25.7 |
| *Circumferential - Apical* |  |  |  |
| Strain (%) | -23.8 ± 3.4 | -25.3 ± 3.2 | 4.6 |
| SR_S_ (s ^-1^) | -1.49 ± 0.24 | -1.62 ± 0.28 | 9.5 |
| SR_E_ (s ^-1^) | 1.81 ± 0.48 | 1.89 ± 0.52 | 15.7 |
| SR_A_ (s ^-1^) | 0.53 ± 0.21 | 0.52 ± 0.22 | 40.8 |
| *Circumferential - Global* |  |  |  |
| Strain (%) | -19.3 ± 2.0 | -20.4 ± 2.6 | 5.8 |
| SR_S_ (s ^-1^) | -1.20 ± 0.13 | -1.25 ± 0.15 | 4.5 |
| SR_E_ (s ^-1^) | 1.47 ± 0.23 | 1.53 ± 0.36 | 10.9 |
| SR_A_ (s ^-1^) | 0.44 ± 0.17 | 0.46 ± 0.11 | 28.3 |
| *Radial - Basal* |  |  |  |
| Strain (%) | 37.2 ± 14.2 | 48.1 ± 18.7 | 38.0 |
| SR_S_ (s ^-1^) | 2.53 ± 0.76 | 2.60 ± 0.72 | 20.8 |
| SR_E_ (s ^-1^) | -2.11 ± 0.72 | -2.77 ± 1.13 | 35.0 |
| SR_A_ (s ^-1^) | -1.36 ± 0.41 | -1.68 ± 1.19 | 38.8 |
| *Radial - Apical* |  |  |  |
| Strain (%) | 33.5 ± 26.4 | 25.4 ± 24.8 | 31.5 |
| SR_S_ (s ^-1^) | 1.96 ± 1.01 | 1.75 ± 0.80 | 20.8 |
| SR_E_ (s ^-1^) | 2.50 ± 1.29 | 2.20 ± 1.19 | 33.3 |
| SR_A_ (s ^-1^) | -0.46 ± 0.36 | -0.69 ± 0.49 | 81.1 |
| *Radial - Global* |  |  |  |
| Strain (%) | 34.1 ± 16.1 | 38.2 ± 20.6 | 22.7 |
| SR_S_ (s ^-1^) | 2.22 ± 0.63 | 2.15 ± 0.48 | 11.7 |
| SR_E_ (s ^-1^) | -2.22 ± 0.82 | -2.59 ± 1.09 | 24.5 |
| SR_A_ (s ^-1^) | -0.85 ± 0.34 | -1.26 ± 0.75 | 34.9 |

Data are mean ± SD. SR_S_, strain rate systole; SR_E_, strain rate early diastole; SR_A_, strain rate late diastole.


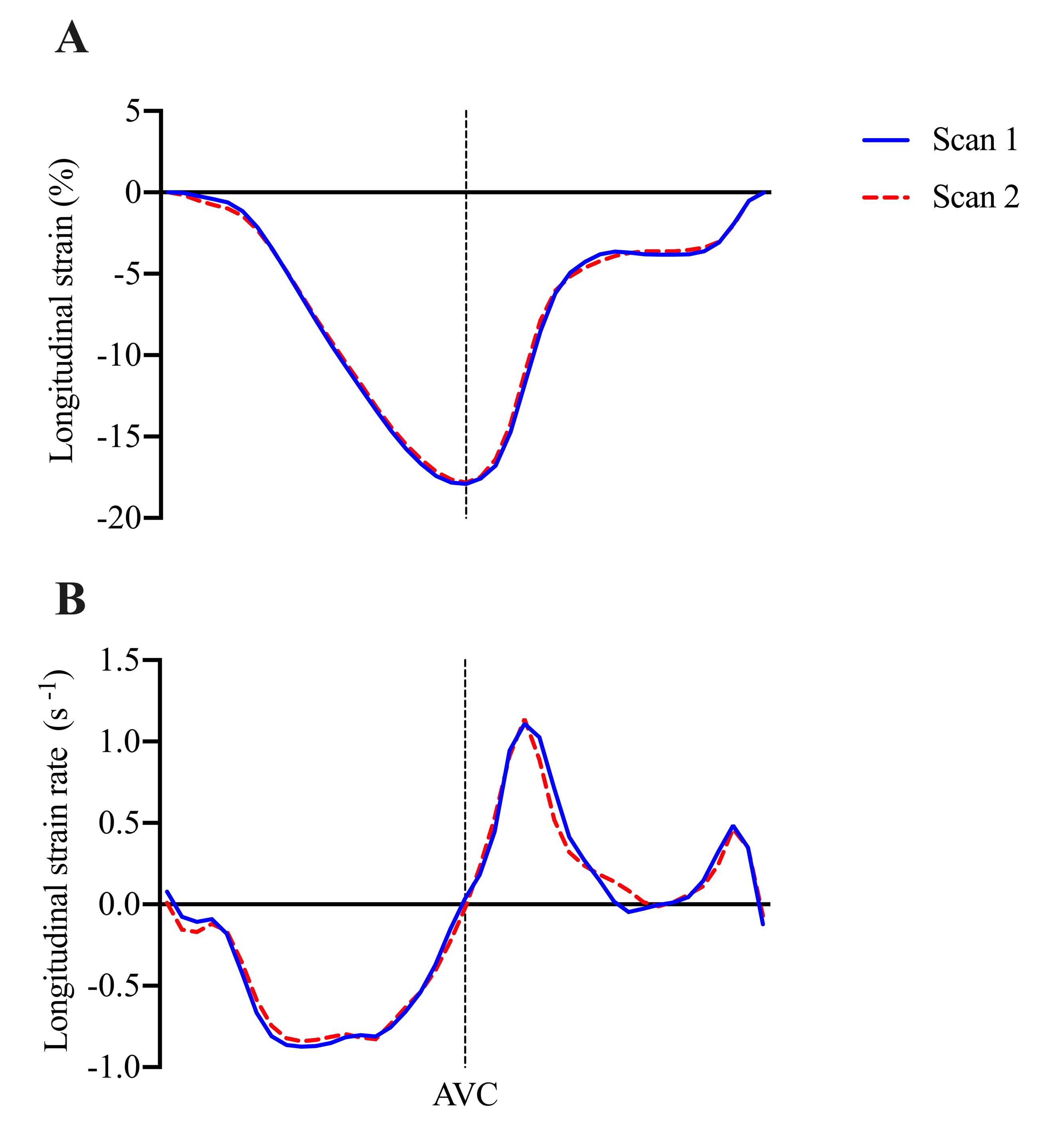


Figure 1. Longitudinal strain (A) and strain rate (B) of scans 1 and 2 across the cardiac cycle in 5% increments. Data are mean values. AVC, aortic valve closure.


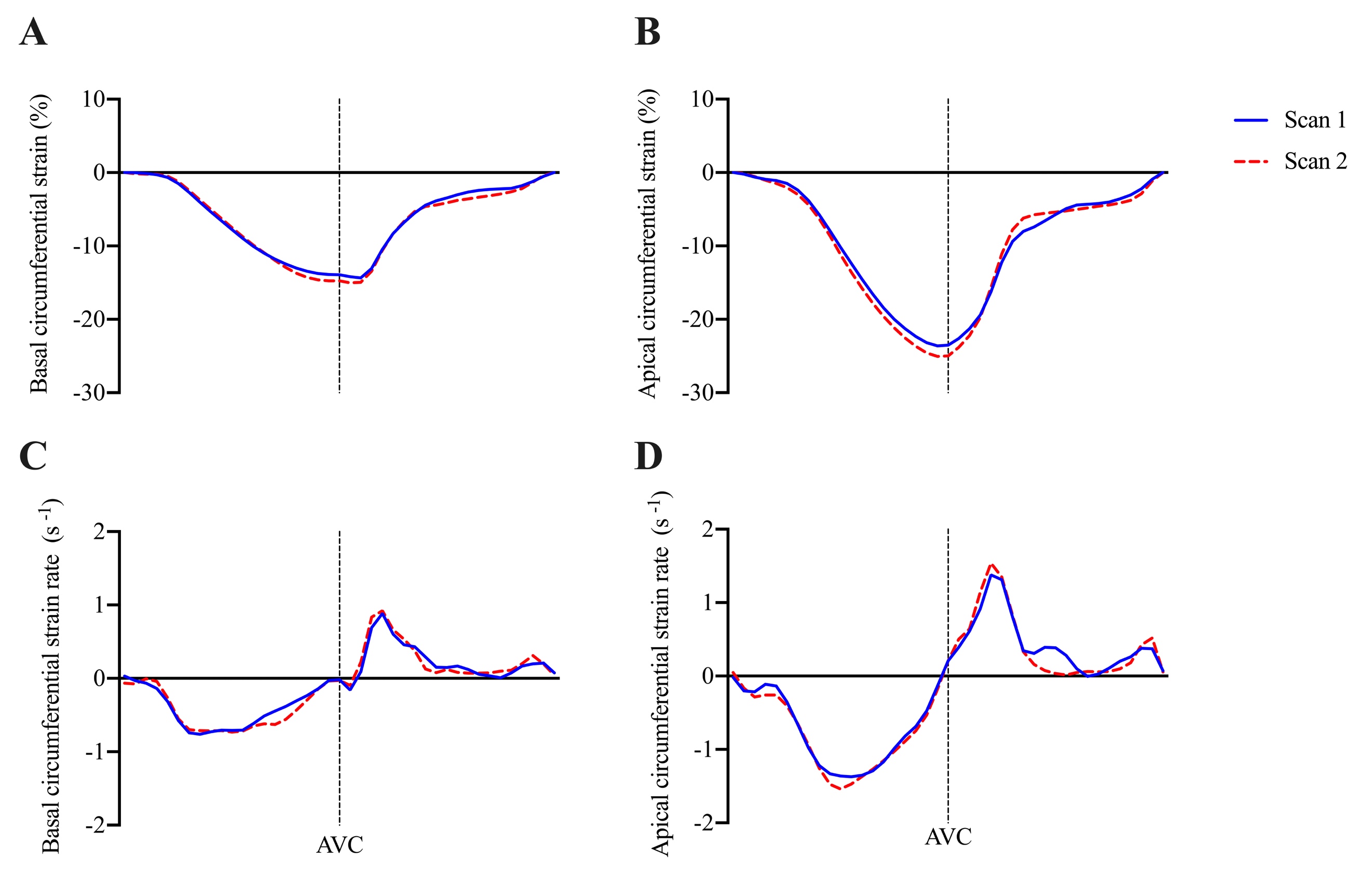


Figure 2. Circumferential strain and strain rate at the base (A, C) and apex (B, C) of scans 1 and 2 across the cardiac cycle in 5% increments. Data are mean values. AVC, aortic valve closure.
